# Capsaicin-Induced Skin Desensitization Differentially Affects A-Delta and C-Fiber-Mediated Heat Sensitivity

**DOI:** 10.3389/fphar.2020.00615

**Published:** 2020-05-19

**Authors:** Sabien G. A. van Neerven, André Mouraux

**Affiliations:** Institute of Neuroscience, Université Catholique de Louvain, Brussels, Belgium

**Keywords:** capsaicin, skin, perception, plasticity, sensory system, pain medication, patch, primary afferent

## Abstract

Localized neuropathic pain can be relieved following the topical application of high-concentration capsaicin. This clinical effect is thought to be related to the temporary desensitization of capsaicin- and heat-sensitive epidermal nociceptors. The objective of the present study was to examine whether the changes in thermal sensitivity induced by high-concentration topical capsaicin can be explained entirely by desensitization of capsaicin-sensitive afferents. For this purpose, we characterized, in 20 healthy human volunteers, the time course and spatial extent of the changes in sensitivity to thermal stimuli preferentially activating heat-sensitive A-fiber nociceptors, heat-sensitive C-fiber afferents, and cool-sensitive A-fiber afferents. The volar forearm was treated with a high-concentration capsaicin patch for 1 h. Transient heat, warm and cold stimuli designed to activate Aδ- and C-fiber thermonociceptors, C-fiber warm receptors, and Aδ-fiber cold receptors were applied to the skin before and after treatment at days 1, 3, and 7. Reaction times, intensity ratings, and quality descriptors were collected. The stimuli were applied both within the capsaicin-treated skin and around the capsaicin-treated skin to map the changes in thermal sensitivity. We found that topical capsaicin selectively impairs heat sensitivity without any concomitant changes in cold sensitivity. Most interestingly, we observed a differential effect on the sensitivity to thermal inputs conveyed by Aδ- and C-fibers. Reduced sensitivity to Aδ-fiber-mediated heat was restricted to the capsaicin-treated skin, whereas reduced sensitivity to C-fiber-mediated heat extended well beyond the treated skin. Moreover, the time course of the reduced sensitivity to C-fiber-mediated input was more prolonged than the reduced sensitivity to Aδ-fiber-mediated input.

## Introduction

High-concentration topical capsaicin application is used to treat localized neuropathic pain. The relief of pain that may follow this topical treatment is thought to be related to the temporary deactivation of heat-sensitive epidermal nociceptors expressing the Transient Receptor Potential Vanilloid 1 (TRPV1) (Caterina et al., 1997; Wang et al., 2017). Skin biopsies conducted after prolonged or high-concentration application of capsaicin onto the skin have shown that it induces a marked reduction of intra-epidermal nerve fiber density (Nolano et al., 1999; Rage et al., 2010). Very recently, Wang et al. (2017) showed that this “axonal ablation” results from TRPV1-mediated calcium influx and the activation of calcium-dependent calpain. In healthy volunteers, topical capsaicin has been used to study the role of TRPV1 in heat perception (Nolano et al., 1999; Polydefkis et al., 2004; Kennedy et al., 2010; Rage et al., 2010; Lo Vecchio et al., 2018) and to investigate the contribution of TRPV1-positive fibers in sensitization and hyperalgesia (Ziegler et al., 1999; Magerl et al., 2001; Henrich et al., 2015). The reduced sensitivity to heat observed after topical capsaicin in healthy volunteers appears to be short lasting, with maximum effects observed 1–3 days after treatment followed by rapid recovery (Rage et al., 2010; Bauchy et al., 2016; Lo Vecchio et al., 2018). In healthy volunteers, a divergence has been reported between the quite rapid return of heat sensitivity and a slower return of visible epidermal nerve endings in skin biopsies (Kennedy et al., 2010; Rage et al., 2010).

Capsaicin is supposed to specifically act on TRPV1-expressing nociceptors in the skin, thereby selectively abolishing sensitivity to noxious temperatures (>46°C) (Treede et al., 1995; Ringkamp et al., 2001; Churyukanov et al., 2012). In rodents, it was shown that TRPV1 is one of three functionally-redundant TRP-receptors (TRPV1, TRPM3, and TRPA1), as the behavioral responses to noxious heat stimuli are abolished only when all three receptors are knocked out (Vandewauw et al., 2018). During the acute phase of topical capsaicin treatment (i.e. during its application and the hours that follow application), a marked hypersensitivity to heat and mechanical pinprick stimuli is observed at the treated area, as well as in the surrounding skin (Culp et al., 1989). Increased heat sensitivity has been ascribed to a direct effect of capsaicin on heat-sensitive TRPV1-expressing nociceptors (Caterina et al., 1997), whereas the increased sensitivity to mechanical pinprick stimuli is thought to be related to secondary hyperalgesia and sensitization at the level of the central nervous system (Henrich et al., 2015). These studies point out that capsaicin-responsive nerve fibers play a crucial role in the transduction and perception of acute thermal stimuli as well as in the adaptation of the central nervous system after harmful events such as noxious temperatures.

Since topical capsaicin is thought to temporarily deactivate a particular population of nerve fibers, those expressing TRPV1, it can be used to study the contribution of capsaicin-sensitive fibers to the perception of newly encountered sensory stimuli and the perceptual adaptation to a temporary loss of function of these fibers. To our knowledge, it was never investigated how sensory fibers in the skin surrounding the capsaicin-denervated area respond to the selective ablation of capsaicin-sensitive fibers. For this reason, in the present study, we performed a sensory mapping of the human skin using different types of thermal stimuli expected to preferentially activate different types of heat-sensitive afferents. Brief high-intensity heat stimuli were used to generate responses predominantly related to the activation of quickly responding type 2 Aδ-fiber heat nociceptors. Short-lasting intermediate- and low-intensity heat stimuli were used to generate responses related to the preferential activation of heat-sensitive C-fiber nociceptors or C-fiber warm receptors, having a lower thermal activation threshold than type 2 Aδ-fiber nociceptors (Hallin et al., 1982; Green and Cruz, 1998; Plaghki and Mouraux, 2003; Wooten et al., 2014). Finally, innocuous cool stimuli were used to produce responses related to the selective activation of cool-sensitive A-fiber afferents (Yarnitsky and Ochoa, 1991; Campero et al., 2001). All stimuli were applied not only within the capsaicin-treated area, but also at the border of the treated area, and at control sites distant from the treated area.

The study aimed to answer the following three questions. First, does topical capsaicin alter only the perception of noxious heat or are other thermal modalities affected? Second, what are the peripheral afferents involved in the reduced thermal sensitivity that follows treatment with topical capsaicin? Third, does capsaicin only affect thermal sensitivity within the treated skin, or does it also affect thermal sensitivity in the surrounding non-treated skin, possibly because the sensory system reorganizes in response to the ablation of capsaicin-sensitive fibers?

## Materials and Methods

### Participants

Twenty-one healthy volunteers were enrolled in this study (11 men and 10 women; aged 22–50 years; 27.0 ± 1.5 years [mean ± SEM]). The experiments were conducted according to the Declaration of Helsinki. Approval for the conduction of the experiment was obtained from the local Ethical Committee (UCLouvain commission d’éthique bio-médicale hospitalo-facultaire). All participants signed an informed consent form and received financial compensation for their participation.

### Forearm Mapping

**Figure 1:**
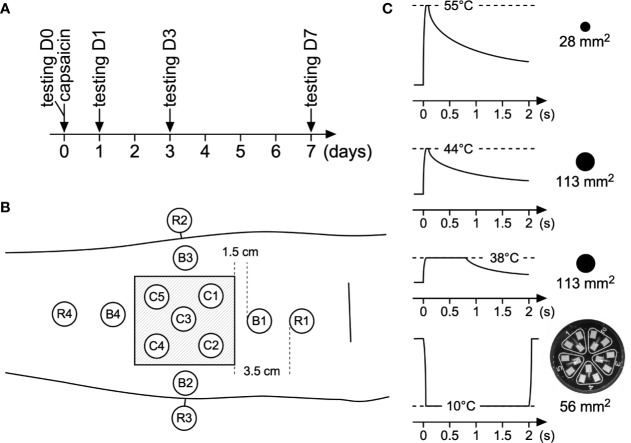
Time line, area stimulated, and sensory stimuli used in this study. **(A)** Sensory testing at baseline was performed on day 0 (D0), after which participants received a capsaicin patch for 1 h. Thereafter post-treatment sensory testing was conducted at days 1, 3, and 7 (D1, D3, D7) after capsaicin treatment. **(B)** Testing locations at the volar forearm: C1–C5: capsaicin-treated skin; B1–B4: border skin (1.5 cm away from the border of capsaicin-treated skin), R1–R4 remote skin (3.5 cm away from the border of capsaicin-treated skin). Additionally four locations at the contralateral arm were stimulated. For each type of stimulus, every single location was stimulated three times. **(C)** Different types of transient thermal stimuli were used to assess capsaicin-induced changes in thermal sensitivity.

With the help of a template applied against the skin, 13 test areas were defined on the capsaicin-treated forearm as shown in **Figure 1**. Five test areas were located inside the 5 × 5-cm area of capsaicin-treated skin (C1–C5), whose center was positioned 8 cm distal from the cubital fossa. Four test areas were located close to the border of the treated skin (B1–B4; edge of test area 1.5 cm from the edge of the capsaicin-treated skin). Four test areas were defined further away from the treated skin, at more remote locations (R1–R4; edge of test area 3.5 cm away from the borders of the capsaicin-treated skin). Finally, four additional test areas were defined on the contralateral forearm, at the locations corresponding to the area of capsaicin application on the capsaicin-treated forearm (CON1–CON4).

### Topical Capsaicin

High-concentration capsaicin patches were made using a 2% solution of capsaicin (capsaicin ≥ 95%, 8-methyl-N-vanillyl-trans-6-nonenamide M2028, Sigma Aldrich) diluted in 50:50 H_2_O/ethanol. It should be noted that Trevisani et al. (2002) found that ethanol potentiates the response of TRPV1 to capsaicin. The use of ethanol as vehicle could thus potentiate the effect of the capsaicin treatment (Trevisani et al., 2002).

Of this solution, 1 ml was dripped onto a 5 × 5-cm gauze (Sterilux^®^ ES, Paul Hartmann AG, Heidenheim, Germany). The patches were then placed onto the skin, and wrapped using a self-adhesive transparent wound bandage (Opsite Flexifix Gentle, Smith & Nephew, Hull, England). The capsaicin patch remained in place for 1 h.

During the application, participants were asked to report the intensity of the capsaicin-induced sensation every 15 min using a 0–100 numerical rating scale (NRS) where 0 was defined as “no pain at all” and 100 was defined as “the maximum imaginable pain.” After treatment, the patch was removed and the arm was gently washed with water and soap to remove capsaicin residuals. Participants were asked to qualify the sensation induced by topical capsaicin by selecting one or more of the following quality descriptors: “not perceived,” “touch,” “tingling,” “pricking,” “pointed,” “warm,” “burning,” “electric shock,” “cool,” “wet,” and “unpleasant.”

### Laser Heat Stimuli

Participants were seated comfortably on a chair, with their arms placed on a cushion on a table, volar forearms facing upwards.

Three different types of heat stimuli were delivered using a temperature-controlled CO_2_ laser stimulator (LSD, SIFEC, Belgium), with two different beam diameters: 6 mm (28 mm^2^) or 12 mm (113 mm^2^). This laser stimulator produces a laser beam with a flat-top rather than a Gaussian power density profile. Power output and, hence, target temperature, is thus homogeneously distributed throughout the stimulated surface. Laser stimuli were delivered perpendicular to the skin. The laser head was maintained above the forearms using a fixation tripod.

The first type of heat stimulus was a high-intensity 55°C stimulus lasting 100 ms and delivered using a 6-mm laser beam diameter. The second type of heat stimulus was an intermediate-intensity 44°C stimulus lasting 100 ms and delivered using a 12-mm beam diameter. The third type of heat stimulus was a low-intensity 38°C stimulus lasting 800 ms and delivered using a 12- mm beam diameter. On normal human skin, the high intensity stimulus can be expected to activate type 2 Aδ-fiber heat nociceptors as studies have shown that their thermal activation threshold to transient heat pulses is in the range of 46–53°C (Treede et al., 1995). Intermediate intensity stimuli can be expected to activate C-fiber thermonociceptors having a thermal activation threshold to transient heat stimuli of approximately 41°C (Wooten et al., 2014) without concomitantly activating Aδ-fiber heat nociceptors. Finally, low-intensity heat stimuli can be expected to selectively activate C-fiber warm receptors. Several previous studies have shown that the high-intensity stimulus used in the present study are detected with reaction times (RT) compatible with the conduction velocity of myelinated Aδ fibers, whereas the intermediate and low intensity stimuli are detected with much slower RTs compatible with the conduction velocity of unmyelinated C-fibers (Churyukanov et al., 2012). A 12-mm beam diameter was used for the intermediate (44°C) and low (38°C) intensity heat stimuli because in pilot experiments conducted for other studies (Churyukanov et al., 2012; Jankovski et al., 2013), we found that increasing beam diameter (12 mm instead of 6 mm) increased the likelihood for such stimuli to generate responses related to the selective activation of C-fiber afferents. The same approach of using a large stimulation surface has been used by other groups to record C-fiber-related laser-evoked responses (Cruccu et al., 2003).

### Contact Cool Stimuli

In addition to assessing sensitivity to heat stimuli, we also assessed sensitivity to transient cool stimuli delivered using a contact thermode (TCS-II, QST.Lab, Strasbourg, France; cooling ramp >200°C/s). The stimulation surface of the thermode (9.6 cm^2^) is divided in five segments, each containing three micro-Peltier elements having a surface of 9.3 mm^2^. The cool stimuli consisted in cooling the skin to 10°C during 2000 ms using two adjacent segments of the five available segments, resulting in a total stimulation surface of 56 mm^2^.

### Mechanical Pinprick Stimuli

Finally, we assessed sensitivity to mechanical pinprick stimuli using a custom made 128-mN probe consisting of a 0.35 mm flat-tip weighted needle mounted in a sliding cylinder (van den Broeke et al., 2016). The device was applied perpendicular to the skin during 1 s and subsequently removed.

### Sensory Testing

Sensory testing was performed at baseline before application of the capsaicin patch (D0), the day after capsaicin treatment (D1), 3 days after treatment (D3), and 7 days after treatment (D7).

The different types of stimuli (55°C heat, 44°C heat, 38°C heat, 10°C cool, and pinprick) were applied in separate blocks (one block per stimulation modality for a total of five blocks), whose order was randomized across participants, except for the high-intensity heat stimulus that was always tested last to avoid skin sensitization from interfering with the other assessments.

Within each block, each testing site of each area was stimulated sequentially (the order of the sequence was randomized across one block). This sequence was repeated three times, resulting in a total of 5 × 3 = 15 stimuli delivered to the capsaicin-treated skin (C1–C5), 4 × 3 = 12 stimuli delivered close to the border of the treated skin (B1–B4), 4 × 3 = 12 stimuli delivered to remote locations of the capsaicin-treated forearm (R1–R4), and 4 × 3 = 12 stimuli delivered to the contralateral forearm (CON1–CON4). Each sequence was followed by a break lasting at least 5 min, and during which the participants were distracted from their task. They were asked for after sensations after each sequence and, in case these were present, the pause was extended until these sensations ceased. Because the testing sequence lasted approximately 10 min, the time interval between two stimuli applied to the same spot was at least 15 min.

For each stimulus, RTs were recorded by asking participants to press a button held in the contralateral hand as soon as they perceived the stimulus. Participants were told to look away from the stimulated forearm and were alerted verbally before stimulus application. Undetected trials were defined as trials that did not produce any reaction or trials detected with RT exceeding 2.5 s.

Immediately after the RT task, participants were asked to rate the intensity of the perceived sensation using a 0–100 NRS where 0 was defined as “no sensation at all”, 100 was defined as “the maximal imaginable intensity or pain” and 50 was defined as delimiting the transition between non-painful and painful domains of sensation. Undetected trials were ascribed an intensity of 0. Average intensity ratings were calculated for each stimulated area and time point. Then, for each stimulated area, intensity ratings were expressed as percentages relative to the average intensity ratings obtained at time point D0 (100% thus corresponds to no change in perception intensity as compared to baseline, and values greater or smaller than 100% correspond to increases and decreases in perception intensity relative to baseline).

Finally, participants were asked to choose one or more quality labels from the following list of descriptors: “not perceived,” “touch,” “tingling,” “pricking,” “pointed,” “warm,” “burning,” “electric shock,” “cool,” “wet,” “unpleasant.” The quality “not perceived” was exclusive, meaning that no other quality descriptors were assigned when stimuli were not detected. As previously mentioned, these trails received intensity rating 0, were counted in the number of non-perceived trials, and excluded from RT calculations. For each tested area and each descriptor of quality, the percentage of positive responses was calculated per total number of stimuli per area. Only the quality ratings that were assigned to more than 20% of total stimuli at baseline are reported.

### Statistical Analysis

All data were tested for normality and equal variances. Data were not normally distributed. NRS scores were tested with a non-parametric one-way ANOVA followed by Friedman’s multiple comparison test. RTs, intensity and quality ratings were tested *via* repeated-measures two-way ANOVAs (mixed-effects model), with factors time (four levels: D0, D1, D3, and D7) and location (four levels: remote, border, center, contralateral), using Tukey’s multiple comparisons post-hoc test. Sphericity was not assumed, as such Geisser–Greenhouse correction was applied. Comparisons were performed across “location” and across “time.” P-values were corrected for post-hoc multiple comparison. P-values below 0.05 were considered as statistically significant.

## Results

### Capsaicin Treatment

**Figure 2:**
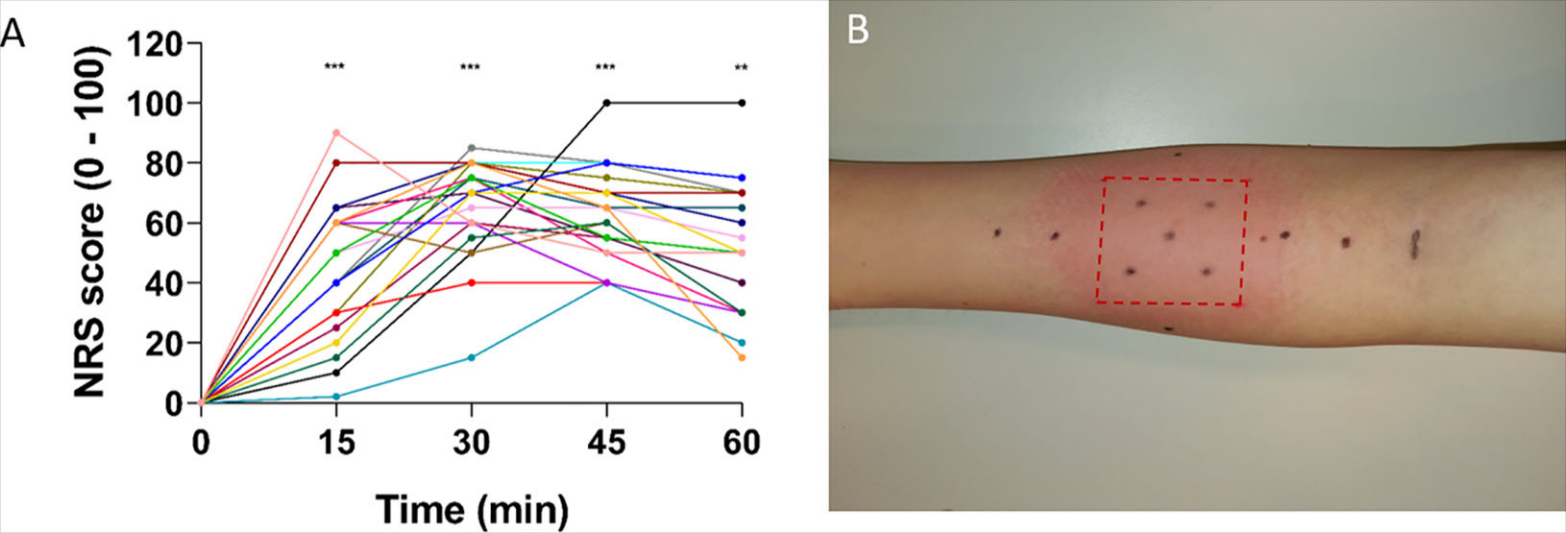
NRS scores during capsaicin treatment and flare response immediately after removal of the patch. **(A)** NRS scores increased after capsaicin treatment with mean scores peaking 30–45 min after application (n = 21). Individual ratings are represented as dots. Values in time were compared to t = 0 (** ≤ 0.01 and *** ≤ 0.001). **(B)** Flare response immediately after patch removal in one representative subject. Red dotted lines indicate the area of the patch. Generally, the flare disappeared within 2–3 h after patch removal. NRS, numerical rating scale.

The intensity of the burning sensation induced by capsaicin application increased from 0 to 46 ± 5.3 over the first 15 min (**Figure 2A**). Intensity was maximal at 30 min (66 ± 3.7, p < 0.001) and then tended to stabilize or slowly descend at 45 min (63 ± 3.3, p < 0.001) and 60 min (50 ± 4.9, p < 0.01). After removal of the patch, a clear cutaneous flare was visible at and around the application site (**Figure 2B**). Participants described the sensation induced by topical capsaicin as “warm,” “pricking,” “burning,” “itching,” and “unpleasant.”

### Sensitivity to Short-Lasting High-Intensity Heat Stimuli (55°C)

**Figure 3:**
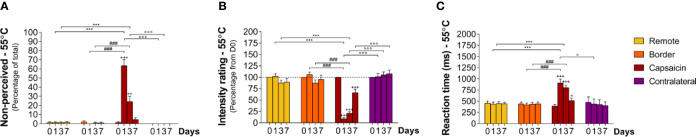
Sensory responding to high-intensity heat stimuli at remote locations of the capsaicin-treated forearm (yellow), close to the border of the capsaicin-treated skin (orange), at the capsaicin-treated skin (red), and at the contralateral forearm (purple). **(A)** Percentage of non-perceived stimuli in response to high-intensity heat stimuli predominantly activating Aδ-fiber thermonociceptors [Aδ-fiber heat (55°C)]. **(B)** Intensity ratings. **(C)** Reaction times. Graphs represent mean ± SEM. The + indicates significant differences at D1–D7 compared to D0 within one location, *indicates significant differences between remote and capsaicin locations, ^#^indicates significant difference between border and capsaicin locations, and °indicates significant differences between contralateral locations compared to the capsaicin-treated location (+/° ≤ 0.05, ++ ≤ 0.01 and ***/+++/###/°°° ≤ 0.001).

At baseline (D0), short-lasting high-intensity heat stimuli were almost always detected; approximately 99% of all stimuli were perceived (non-perceived stimuli: capsaicin: 1 ± 1.0%, border: 2 ± 1.1%, remote: 1 ± 1.0%, and contralateral: 0%, **Table 1**) at all locations (**Figures 3A, B**) with rapid RTs (e.g. 392 ± 34 ms at C1–C5 locations) compatible with the conduction velocity of myelinated Aδ-fiber thermonociceptors (**Figures 3C** and **7**; **Table 1**). The three descriptors most-often chosen by the participants to qualify the elicited sensation were “pricking,” “burning,” and “unpleasant” (**Supplementary Figure S1**).

**Table 1:** Percentage of non-perceived stimuli, intensity ratings, and reaction time latencies at baseline (D0) and statistical analysis of the data-sets by repeated-measures two-way ANOVAs (mixed-effects model), with factors time (four levels: D0, D1, D3, and D7) and location (four levels: remote, border, center, contralateral), using Tukey’s multiple comparisons post-hoc test.

Percentage non-perceived	High-intensity heat (55°C)	Intermediate-intensity heat (44°C)	Low-intensity heat (38°C)	Cool (10°C)

**Percentage non-detected at D0**				
- Capsaicin area	1 ± 1.0%	12 ± 4.0%	13 ± 3.6%	1 ± 0.9%
- Border area	2 ± 1.1%	8 ± 3.1%	14 ± 4.9%	3 ± 1.2%
- Remote area	1 ± 1.0%	14 ± 4.0%	19 ± 4.0%	2 ± 1.2%
- Contralateral area.	0%	11 ± 4.1%	11 ± 6.1%	0%
**Main effect of time**	F_3,60_ = 21.7	F_3,176_ = 38.8	F_2,99_ = 27.2	F_2,134_ = 0.5
**Main effect of location**	F_3,60_ = 48.6	F_3,62_ = 20.9	F_3,43_ = 36.7	F_3,62_ = 1.1
**Time × location interaction**	F_9,120_ = 36.5	F_9,186_ = 21.7	F_9,129_ = 11.5	F_9,186_ = 0.6
**Intensity of perception**				
**Intensity of perception at D0**				
- Capsaicin area	55 ± 3.7	15 ± 2.3	9 ± 1.4	16 ± 1.6
- Border area	52 ± 3.9	15 ± 1.9	10 ± 1.5	15 ± 1.6
- Remote area	52 ± 3.7	13 ± 1.5	8 ± 1.0	15 ± 1.5
- Contralateral area	60 ± 5.9	14 ± 1.6	11 ± 0.8	19 ± 2.9
**Main effect of time**	F_2,134_ = 16.9	F_3,161_ = 56.4	F_3,112_ = 36.4	F_2,119_ = 4.3
**Main effect of location**	F_3,65_ = 35.3	F_3,62_ = 11.0	F_3,43_ = 11.4	F_3,62_ = 0.02
**Time × location interaction**	F_9,195_ = 24	F_9,186 =_ 5.4	F_9,129_ = 4.8	F_9,186 =_ 0.07
**Reaction time latencies**				
**Reaction times at D0** - Capsaicin area	392 ± 34 ms	850 ± 69 ms	900 ± 64 ms	560 ± 65 ms
- Border area	441 ± 44 ms	820 ± 42 ms	879 ± 54 ms	534 ± 43 ms
- Remote area	458 ± 47 ms	809 ± 47 ms	997 ± 77 ms	572 ± 52 ms
- Contralateral area	476 ± 119 ms	849 ± 79 ms	971 ± 143 ms	540 ± 68 ms
**Main effect of time**	F_2,137_ = 10.7	F_2,147_ = 4.1	F_3,100_ = 4.0	F_3,173_ = 4.9
**Main effect of location**	F_3,65_ = 5.8	F_3,62_ = 1.8	F_3,43_ = 2.5	F_3,65_ = 0.1
**Time × location interaction**	F_9,192_ = 20.6	F_9,174_ = 1.3	F_9,121_ = 2.9	F_9,192_ = 0.6

After topical capsaicin, the detection of high-intensity heat stimuli was markedly impaired at the treated skin (C1–C5), but not at the border of the treated skin (B1–B4), at remote skin of the treated forearm (R1–R4), and at the contralateral forearm (**Figure 3B**; **Tables 2** and **3**). Loss of sensitivity within the treated skin was maximal 24 h after capsaicin treatment (D1), with 64 ± 6.7% (p < 0.001) of stimuli delivered to the treated skin being undetected (**Figure 3A**; **Tables 2** and **3**) and intensity ratings dropping to 9 ± 3.0% (p < 0.001) of the ratings provided at D0 (**Figure 3B**; **Tables 2** and **3**). Furthermore, the average RT to detected stimuli (909 ± 87 ms) was markedly lengthened as compared to D0 (**Figure 3C**; **Tables 2** and **3**; p < 0.001). Three days after capsaicin application (D3), 24 ± 5.9% of the stimuli remained undetected (p < 0.001), and mean intensity ratings were still reduced (21 ± 3.4% relative to D0, p < 0.001). Such as at D1, average RTs of detected stimuli (804 ± 56 ms) were increased as compared to D0 (p < 0.001). Seven days after capsaicin treatment (D7), almost all stimuli were detected, with only 5 ± 1.9% undetected stimuli. Nevertheless, intensity ratings were still reduced when compared to baseline (66 ± 7.3% relative to D0, < 0.001). Likewise, RTs of detected stimuli remained moderately increased (515 ± 56 ms, **Figures 3C** and **7**; **Tables 2** and **3**; p < 0.05).

**Table 2:** Comparisons of the percentage of non-perceived stimuli, intensity ratings, and reaction times across sites.

Percentage of non-perceived stimuli		Capsaicin vs. border area	Capsaicin vs. remote area	Capsaicin vs. contralateral area

**High-intensity heat (55°C)**	**D0**	T = 0.714 p = 0.997	T = −0.081 p > 0.9999	T = 0.436 p = 0.9998
	**D1**	T = −63.49 p < 0.0001^***^	T = −62.30 p < 0.0001^***^	T= 62.59 p < 0.0001^***^
	**D3**	T = −23.33 p < 0.0001^***^	T = −22.94 p < 0.0001^***^	T = 2315 p = 0.0002^***^
	**D7**	T = −3.652 p = 0.7350	T = −2.867 p = 0.8522	T = 3.599 p = 0.9105
**Intermediate-intensity heat (44°C)**	**D0**	T = −4.425 p = 0.8172	T = 1.410 p = 0.9945	T = 1.240 p = 0.9962
	**D1**	T = −61.92 p < 0.0001^***^	T = −66.08 p < 0.0001^***^	T = 72.35 p < 0.0001^***^
	**D3**	T = −54.91 p < 0.0001^***^	T = −54.09 p < 0.0001^***^	T = 58.68 p < 0.0001^***^
	**D7**	T=−14.01 p = 0.1894	T = −6.080 p = 0.8462	T = 18.09 p = 0.0847
**Low-intensity heat (38°C)**	**D0**	T = 1.671 p = 0.9925	T = 5.843 p = 0.7005	T = 1.507 p = 0.9962
	**D1**	T = 48.45 p < 0.0001^***^	T =−4844 p < 0.0001^***^	T = 63.42 p < 0.0001^***^
	**D3**	T = −53.69 p < 0.0001^***^	T = −53.09 p < 0.0001^***^	T = 65.09 p < 0.0001^***^
	**D7**	T = −16.54 p = 0.3054	T = −14.16 p = 0.3847	T = 2557 p = 0.0168^*^
**Cool (10°C)**	**D0**	T = 1.170 p = 0.8696	T = 0.7550 p = 0.9580	T = 1.330 p = 0.4837
	**D1**	T = −3.010 p = 0.4353	T = −3.005 p = 0.4744	T = 4.670 p = 0.0800
	**D3**	T = −0.675 p = 0.9918	T = −0.675 p = 0.9918	T= 4.005 p = 0.0624
	**D7**	T = −2.340 p = 0.8064	T = −3.175 p = 0.5858	T= 4.010 p = 0.3449
**Intensity of perception % from D0**				
**High**-**intensity heat (55°C)**	**D1**	T = 97.21 p < 0.0001^***^	T = 93.88 p < 0.0001^***^	T = −92.65 p < 0.0001^***^
	**D3**	T = 66.10 p < 0.0001^***^	T = 66.33 p < 0.0001^***^	T = −84.26 p < 0.0001^***^
	**D7**	T = 29.06 p = 0.0904	T = 23.14 p = 0.1423	T = −41.44 p = 0.0067^***^
**Intermediate**-**intensity heat (44°C)**	**D1**	T = 44.91 p < 0.0001^***^	T = 44.06 p < 0.0001^***^	T = −77.34 p = 0.0278^***^
	**D3**	T = 43.97 p < 0.0001^***^	T = 46.15 p < 0.0001^***^	T = −55.27 p = 0.0093^***^
	**D7**	T = 22.85 p = 0.3781	T = 9.870 p = 0.6694	T = −34.45 p = 0.4091
**Low**-**intensity heat (38°C)**	**D1**	T = 42.39 p = 0.0004^***^	T = 56.61 p = 0.0006^***^	T = −92.90 p = 0.0059^***^
	**D3**	T = 42.71 p = 0.0011^***^	T = 44.73 p = 0.0023^***^	T = −76.57 p = 0.0439^***^
	**D7**	T = 20.52 p = 0.5878	T = 19.41 p = 0.3410	T = −30.96 p = 0.1645
**Cool (10°C)**	**D1**	T = −3.320 p = 0.9991	T = −3.885 p = 0.9987	T = −4.592 p = 0.9975
	**D3**	T = −1.235 p = 0.9997	T = −0.675 p > 0.9999	T = −9.125 p = 0.9241
	**D7**	T = −1.095 p = 0.9999	T = 1.975 p = 0.9994	T = 4.933 p = 0.9883
**Reaction times**				
**High**-**intensity heat (55°C)**	**D0**	T = 48.34 p = 0.8188	T = 65.68 p = 0.6685	T = −83.94 p = 0.9018
	**D1**	T = −490.1 p = 0.0002^***^	T = −474.1 p = 0.0003^***^	T = 470.3 p = 0.0277^*^
	**D3**	T = −368.6 p < 0.0001^***^	T = −357.7 p < 0.0001^***^	T = 375.1 p = 0.0547
	**D7**	T = −72.26 p = 0.7275	T = −70.67 p = 0.7062	T = 109.2 p = 0.6945
**Intermediate**-**intensity heat (44°C)**	**D0**	T = −29.79 p = 0.9829	T = −40.94 p = 0.9610	T = 0.8117 p > 0.9999
	**D1**	T = −173.7 p = 0.2955	T = −189.0 p = 0.2240	T = 218.8 p = 0.1957
	**D3**	T = −118.0 p = 0.4049	T = −133.8 p = 0.2854	T = 121.0 p = 0.6773
	**D7**	T = −46.68 p = 0.7724	T = −69.88 p = 0.3470	T = 19.49 p = 0.9908
**Low**-**intensity heat (38°C)**	**D0**	T = −20.82 p = 0.9944	T = −96.92 p = 0.7655	T = −71.11 p = 0.9661
	**D1**	T = −353.9 p = 0.2281	T = −384.6 p = 0.1786	T = 355.3 p = 0.2087
	**D3**	T = −73.81 p = 0.8924	T = −131.4 p = 0.5672	T = 50.97 p = 0.9625
	**D7**	T = −129.6 p = 0.1933	T = −146.8 p = 0.0869	T = 95.83 p = 0.7095
**Cool (10°C)**	**D0**	T = −25.59 p = 0.9875	T = 12.07 p = 0.9989	T = 20.21 p = 0.9963
	**D1**	T =−56.90 p = 0.8951	T = −42.84 p = 0.9574	T = 76.33 p = 0.8315
	**D3**	T = −5.225 p = 0.9998	T = 7.420 p = 0.9994	T = 35.06 p = 0.9666
	**D7**	T = 7.348 p = 0.9992	T = 11.50 p = 0.9967	T = −27.72 p = 0.9862

Tables reflect differences in mean and corrected p-values (*indicates p ≤ 0.05 and ***≤ 0.001).

**Table 3:** Comparisons of the percentage of non-perceived stimuli, intensity ratings, and reaction times across time points.

Percentage of non-perceived stimuli		D1 vs. D0	D3 vs. D0	D7 vs. D0
**High**-**intensity heat (55°C)**	Capsaicin area	T = −62.22 p < 0.0001^***^	T = −22.85 p = 0.0034^**^	T = −3.176 p = 0.1198
	Border area	T = 1.986 p = 0.3279	T= 1.190 p = 0.5327	T = 1.190 p = 0.8423
	Remote area	T = 0.0000 p > 0.9999	T = 0.0047 p > 0.9999	T = −0.3905 p = 0.9722
	Contralateral area	T = 0.0000 p > 0.9999	T = 0.0000 p > 0.9999	T = 0.0000 p > 0.9999
**Intermediate**-**intensity heat (44°C)**	Capsaicin area	T = −76.66 p < 0.0001^***^	T = −62.99 p < 0.0001^***^	T = −15.00 p = 0.0217^*^
	Border area	T = −19.16 p = 0.0166^*^	T = −12.51 p = 0.0018^**^	T = −5.415 p = 0.2271
	Remote area	T = −9.165 p = 0.0356^*^	T = −7.495 p = 0.0462^*^	T = −7.510 p = 0.4401
	Contralateral area	T = −5.550 p = 0.5181	T = −5.550 p = 0.7567	T = 1.850 p = 0.9837
**Low**-**intensity heat (38°C)**	Capsaicin area	T = −68.57 p < 0.0001^***^	T = −70.24 p < 0.0001^***^	T = −30.72 p = 0.0038^**^
	Border area	T = −18.45 p = 0.0017^**^	T = −14.88 p = 0.0193^*^	T = −12.51 p = 0.4199
	Remote area	T = −14.29 p = 0.0001^***^	T = −11.31 p = 0.0176^*^	T = −10.72 p = 0.0495^*^
	Contralateral area	T = −6.660 p > 0.9999	T = −6.660 p > 0.9999	T = −6.660 p > 0.9999
**Cool (10°C)**	Capsaicin area	T = −3.340 p = 0.3502	T = −2.675 p = 0.1159	T = −2.680 p = 0.6943
	Border area	T = 0.8400 p = 09220	T = −08300 p = 09715	T = 0.8300 p = 0.9245
	Remote area	T = 0.4200 p = 0.9927	T = −1.245 p = 0.9051	T = 1.250 p = 0.5325
	Contralateral area	T = 0.0000 p > 0.9999	T = 0.0000 p > 0.9999	T = 0.0000 p > 0.9999
**Intensity of perception**				
	
**High**-**intensity heat (55°C)**	Capsaicin area Border area	T = 91.26 p < 0.0001^***^ T = −5.952 p = 0.6746	T = 78.94 p < 0.0001^***^ T = 12.85 p = 0.2631	T = 33.74 p = 0.0008^***^ T = 4.681 p = 09605
	Remote area	T = −2.614 p = 0.9508	T = 12.61 p = 0.2150	T = 1060 p = 05222
	Contralateral area	T = −1.383 p = 0.9963	T = −5.317 p = 0.8357	T = −7.700 p = 0.7554
**Intermediate**-**intensity heat (44°C)**	Capsaicin area	T = 92.76 p < 0.0001^***^	T = 89.72 p < 0.0001^***^	T = 59.54 p < 0.0001^***^
	Border area	T = 47.85 p < 0.0001^***^	T = 45.75 p < 0.0001^***^	T = 36.69 p = 0.0351^*^
	Remote area	T = 48.71 p < 0.0001^***^	T = 43.57 p = 0.0002^***^	T = 49.67 p < 0.0001^***^
	Contralateral area	T = 15.42 p = 0.8315	T = 34.45 p = 0.0791	T = 25.08 p = 0.6065
**Low**-**intensity heat (38°C)**	Capsaicin area	T = 93.32 p < 0.0001^***^	T = 93.21 p < 0.0001^+++^	T = 59.58 p < 0.0001^***^
	Border area	T = 50.93 p < 0.0001^***^	T = 50.50 p = 0.0003^***^	T = 39.06 p = 0.0694
	Remote area	T = 36.71 p = 0.0181^*^	T = 48.48 p = 0.0012^++^	T = 40.16 p = 0.0020^**^
	Contralateral area	T = 0.4200 p > 0.9999	T = 16.64 p = 0.8014	T = 28.62 p = 0.1738
**Cool (10°C)**	Capsaicin area	T = −20.13 p = 0.7243	T = 7.175 p = 0.9183	T = 0.7500 p = 0.9999
	Border area	T = −16.81 p = 0.7130	T = 8.410 p = 0.6711	T = 1.845 p = 0.9979
	Remote area	T = −16.24 p = 0.7610	T = 7.850 p = 0.8231	T = −1.225 p = 0.9996
	Contralateral area	T = −24.72 p = 0.4252	T = −1.950 p = 0.9965	T = 5.683 p = 0.9411
**Reaction times**				
	
**High**-**intensity heat (55°C)**	Capsaicin area	T = −516.5 p < 0.0001^***^	T = −411.7 p < 0.0001^***^	T = −122.2 p = 0.0125^*^
	Border area	T = 21.94 p = 0.6487	T = 5.295 p = 0.9960	T = −1586 p = 0.9997
	Remote area	T = 23.24 p = 0.6965	T = 11.71 p = 0.9195	T = 14.16 p = 0.9439
	Contralateral area	T = 37.77 p = 0.1368	T = 47.35 p = 0.3291	T = 70.93 p = 0.3571
**Intermediate**-**intensity heat (44°C)**	Capsaicin area	T = −132.5 p = 0.4135	T = −43.75 p = 0.8981	T = 60.28 p = 0.6444
	Border area	T = 11.38 p = 0.9889	T = 44.42 p = 0.6323	T = 77.17 p = 0.1427
	Remote area	T = 15.55 p = 0.9887	T = 49.08 p = 0.6701	T = 89.22 p = 0.1114
	Contralateral area	T = 85.43 p = 0.4508	T = 76.40 p = 0.1633	T = 78.95 p = 0.4221
**Low**-**intensity heat (38°C)**	Capsaicin area	T = −437.4 p = 0.0694	T = −1201 p = 0.7100	T = −132.2 p = 0.2049
	Border area	T = −104.4 p = 0.2112	T = −67.11 p = 0.6458	T = −23.36 p = 0.8870
	Remote area	T = 44.05 p = 0.8609	T = 108.2 p = 0.4350	T = 111.6 p = 0.2718
	Contralateral area	T = −11.00 p = 0.9997	T = 1.980 p > 0.9999	T = 34.76 p = 0.9839
**Cool (10°C)**	Capsaicin area	T = −28.17 p = 0.8682	T = 50.14 p = 0.4834	T = 80.92 p = 0.2891
	Border area	T = 3.148 p = 0.9993	T = 29.78 p = 0.7242	T = 47.99 p = 0.2396
	Remote area	T = 26.75 p = 0.4076	T = 54.79 p = 0.1856	T = 81.49 p = 0.1240
	Contralateral area	T = 27.95 p = 0.8004	T = 64.98 p = 0.0928	T = 32.98 p = 0.4593

The table reflects the differences in mean and corrected p-values (* indicates p ≤ 0.05, ** ≤ 0.01, and ***≤ 0.001).

Capsaicin treatment also affected the quality of the sensation elicited by stimulation of the treated skin, without affecting the quality of the sensations elicited by stimulation of the untreated skin. Most prominent, stimuli that were detected at D1 and D3 were no longer reported as “unpleasant” (**Supplementary Figure S1**).

### Sensitivity to Short-Lasting Intermediate-Intensity Heat Stimuli (44°C)

**Figure 4:**
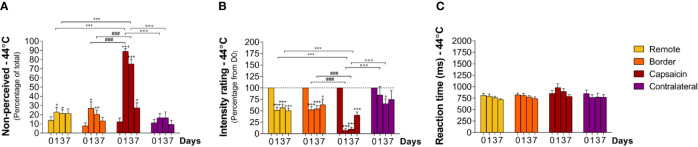
Responding to intermediate-intensity heat stimuli at remote locations of the capsaicin-treated forearm (yellow), close to the border of the capsaicin-treated skin (orange), at the capsaicin-treated skin (red), and at the contralateral forearm (purple). **(A)** Percentage of non-perceived stimuli in response to intermediate-intensity heat stimuli predominantly activating C-fiber thermonociceptors [C-fiber heat (44°C)]. **(B)** Intensity ratings. **(C)** Reaction times. Graphs represent mean ± SEM. The ^+^indicates significant differences at D1–D7 compared to D0 within one location, *indicates significant differences between remote and capsaicin locations, ^#^indicates significant difference between border and capsaicin locations, and ^°^indicates significant differences between the contralateral locations compared to the capsaicin-treated location (+ ≤ 0.05, ++ ≤ 0.01 and ***/+++/###/°°° ≤ 0.001).

At baseline (D0), intermediate-intensity heat stimuli were usually detected (~86% perceived; non-perceived stimuli: capsaicin: 12 ± 4.0% border: 8 ± 3.1% remote: 1 ± 1.0%, and contralateral: 11 ± 4.1%). RTs to these stimuli (e.g. at C1–5: 850 ± 69 ms) were markedly delayed as compared to the RTs to high-intensity heat stimuli (e.g. at C1–5: 392 ± 34 ms), compatible with our expectations that such stimuli would predominantly elicit sensations related to the activation of unmyelinated C-fiber thermonociceptors (**Figures 4C** and **7**; **Tables 2** and **3**). The descriptor most-often chosen by the participants to qualify the elicited sensation was “warm,” sometimes “pricking” and “pointed.” A few times unpleasantness of the stimuli was reported (**Supplementary Figure S2**).

After capsaicin, the number of non-perceived stimuli was 89 ± 3.0% at D1 (relative to D0, p < 0.001), 75 ± 5.2% at D3 (relative to D0, p < 0.001) and 27 ± 5.6% at D7 (relative to D0, p < 0.05), respectively (**Figure 4A**; **Tables 2** and **3**) at the capsaicin-treated area. Intensity ratings dropped massively at D1 (7 ± 3.5% relative to D0, p < 0.001) and D3 (10 ± 3.9% relative to D0, p < 0.001), and remained markedly reduced at D7 (40 ± 6.7% relative to D0, p < 0.001; **Figure 4B**; **Tables 2** and **3**). RTs of the still-perceived stimuli were somewhat increased at D1 relative to D0 (D0: 850 ± 69 ms vs D1: 983 ± 84, n.s., **Figures 4C** and **7**; **Tables 2** and **3**). In contrast, RTs at D3 and D7 were similar to the RTs at D0 (D3: 894 ± 66 ms and D7: 790 ± 37 ms) at the capsaicin-treated area.

Most interestingly, capsaicin treatment impaired the detection of intermediate-intensity heat stimuli not only at the capsaicin-treated skin, but also at the border sites and even at the remote sites of the same forearm. As such, the percentages of non-perceived stimuli were at the remote site 23 ± 5.0% at D1 (relative to D0, p < 0.05), 21 ± 4.2% at D3 (relative to D0, p < 0.05), and 21 ± 4.9% at D7, and at the border site 27 ± 6.9% at D1 (relative to D0, p < 0.05), 20 ± 4.7% at D3 (relative to D0, p < 0.01), and 13 ± 3.9% at D7, respectively (**Figure 4A**; **Tables 2** and **3**). Intensity ratings were lowered relative to baseline values at the remote sites 52 ± 6.1% at D1 (p < 0.001), 56 ± 8.0% at D3 (p < 0.001), and 50 ± 5.5% at D7 (p < 0.001) and at the border sites 52 ± 7.0% at D1 (p < 0.001), 54 ± 7.3% at D3 (p < 0.001), and 63 ± 12.3% at D7 (p < 0.05) (**Figure 4B**; **Tables 2** and **3**).

At the contralateral forearm, no significant changes in perception were observed. Quality of the stimuli did not change much in time, as such, most of the intermediate-intensity stimuli were not perceived at all or were perceived as “warm” (**Supplementary Figure S2**).

### Sensitivity to Low-Intensity Heat Stimuli (38°C)

**Figure 5:**
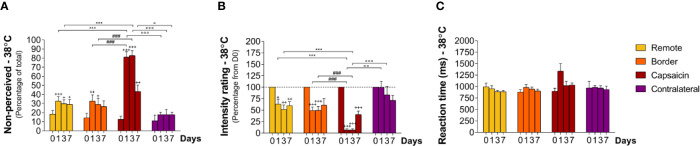
Responding to low-intensity heat stimuli at remote locations of the capsaicin-treated forearm (yellow), close to the border of the capsaicin-treated skin (orange), at the capsaicin-treated skin (red), and at the contralateral forearm (purple). **(A)** Percentage of non-perceived stimuli in response to low-intensity heat stimuli preferentially activating C-warm afferents [C-warm (38°C)]. **(B)** Intensity ratings. **(C)** Reaction times. Graphs represent mean ± SEM. The + indicates significant differences at D1–D7 compared to D0 within one location, ^*^indicates significant differences between remote and capsaicin locations, ^#^indicates significant difference between border and capsaicin locations, and ° indicates significant differences between the contralateral locations compared to the capsaicin-treated location (+/° ≤ 0.05, ++/°° ≤ 0.01 and ***/+++/###/°°° ≤ 0.001).

At baseline (D0), approximately 80% of the low-intensity heat stimuli were detected (**Figure 5A** non-perceived stimuli: capsaicin: 13 ± 3.6% border: 14 ± 4.9% remote: 19 ± 4.0%, and contralateral: 11 ± 6.1%).) and in 1/3 of the participants these low-intensity heating stimuli were not detectable at all (data sets excluded). RTs to these stimuli (e.g. at C1–5 900 ± 64 ms) were compatible with our expectations that such stimuli would predominantly elicit sensations related to the activation of unmyelinated C-warm receptors (**Figures 3C** and **7**; **Tables 2** and **3**). The descriptor most-often chosen by the participants to qualify the elicited sensation was “warm” and stimuli where never “unpleasant” (**Supplementary Figure S3**).

After capsaicin, the number of undetected stimuli was 81 ± 5.8% at D1 (relative to D0 p < 0.001), 83 ± 5.5% at D3 (relative to D0 p < 0.001), and 43 ± 7.0% at D7 (relative to D0 p < 0.01) at the capsaicin-treated area, respectively (**Figure 5A**; **Tables 2** and **3**). Intensity ratings were strongly reduced at D1 (7 ± 2.8% relative to D0, p < 0.001) and D3 (7 ± 2.6% relative to D0, p < 0.001), and remained markedly reduced at D7 (40 ± 7.5% relative to D0, p < 0.001) at the capsaicin-treated area (**Figure 5B**; **Tables 2** and **3**). RTs of the still-perceived stimuli were 1337 ± 162 ms at D1, 1020 ± 95 ms at D3 and 1032 ± 48 ms at D7, compatible with the conduction velocity of unmyelinated C fibers (**Figures 5C** and **7**; **Tables 2** and **3**).

Such as for intermediate-intensity heat stimuli, capsaicin treatment impaired the detection of innocuous low-intensity heat stimuli not only inside the capsaicin-treated skin, but also at border sites and even at the remote sites of the same forearm (**Figures 5A, B**; **Tables 2** and **3**). At the border and remote sites of the capsaicin-treated forearm, the percentage of non-detected stimuli tended to increase at D1 (border: 33 ± 6.7%, relative to D0 p < 0.01; remote: 33 ± 4.9%, relative to D0 p < 0.001), at D3 (border: 29 ± 6.8%, relative to D0 p < 0.05; remote: 30 ± 4.3%, relative to D0 p < 0.05) and at D7 (border: 27 ± 6.1%, relative to D0 n.s.: remote, 29 ± 5.2%, relative to D0 p < 0.05) as compared to D0 (D0: border: 14 ± 4.9%; remote: 18 ± 4.0%). Furthermore, at all three post-capsaicin time-points (D1, D3, and D7) intensity ratings were markedly reduced to approximately 50% of the ratings reported at D0 (**Figure 5B**; **Tables 2** and **3**; remote: D1: 63 ± 10.5%, relative to D0 p < 0.05; D3: 52 ± 9.7%, relative to D0 p < 0.01; D7: 60 ± 8.5% relative to D0 p < 0.01 and border: D1: 49 ± 7.7% relative to D0 p < 0.001; D3: 50 ± 8.5% relative to D0 p < 0.001; D7: 61 ± 14.2% n.s.)

No similar change in perception was observed at the contralateral forearm (**Figure 5B**; **Tables 2** and **3**). Moreover, the stimuli that were perceived were still qualified as being “warm” and were never rated as “unpleasant.”

### Sensitivity to Cool Stimuli (10°C)

**Figure 6:**
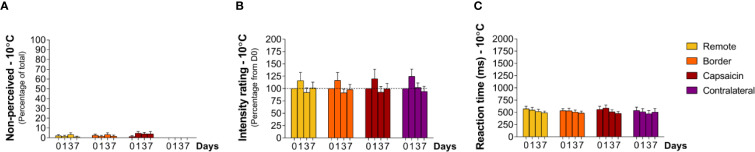
Responding to cooling stimuli at remote locations of the capsaicin-treated forearm (yellow), close to the border of the capsaicin-treated skin (orange), at the capsaicin-treated skin (red), and at the contralateral forearm (purple). **(A)** Percentage of non-perceived stimuli in response to cooling stimuli [Aδ-fiber cold (10°C)]. **(B)** Intensity ratings. **(C)** Reaction times. Graphs represent mean ± SEM.

**Figure 7:**
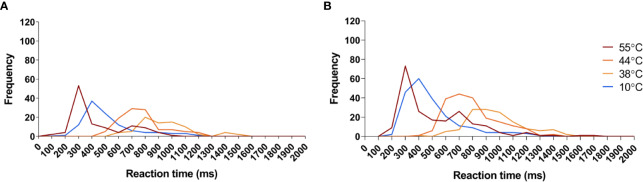
Reaction time frequency distribution of all sensed stimuli in response to the temperatures applied in this study. **(A)** Frequency distribution of reaction times detected before capsaicin treatment. **(B)** Frequency distribution of reaction times detected after capsaicin treatment. Red: Reaction times to 55°C stimuli. Dark orange: reaction times to 44°C stimuli. Orange: reaction times to 38°C stimuli. Blue: reaction times to 10°C stimuli.

At baseline (D0), cool stimuli were usually detected (non-perceived stimuli: capsaicin: 1 ± 0.9% border: 3 ± 1.2% remote: 2 ± 1.2% contralateral: 0%, **Table 1**). RTs to these stimuli (total average: 517 ± 13.3 ms) were compatible with detections related to the activation of cool-sensitive Aδ-fiber afferents (**Figures 6C** and **7**). The two descriptors most-often chosen by the participants to qualify the elicited sensation were “cool” and “wet” (**Supplementary Figure S4**).

Contrasting with the effects of capsaicin treatment on the ability to perceive heat stimuli, the cool sensation elicited by the 10°C stimuli was not significantly affected by capsaicin treatment. At all tested areas, the percentage of detections, intensity ratings and RTs were not significantly changed at D1, D3, and D7 (**Figure 6**).

### Sensitivity to Mechanical Pinprick Stimuli

At baseline (D0), mechanical stimuli were systematically detected (**Supplementary Figure S5**, at all area 0% non-perceived). The descriptors most-frequently chosen by the participants to qualify the elicited sensation were “touch,” “pricking,” and “pointed.” Only rarely, these pinpricks were rated as “unpleasant” at baseline (**Supplementary Figure S5F**).

After capsaicin treatment, sensitivity to 128 mN pinprick stimuli was not affected significantly by capsaicin treatment at any time point, or at any location (**Supplementary Figure S5**). Moreover, the remote effects on sensitivity seen with the intermediate and low-intensity heat stimuli were not detectable for the pinprick stimulations. Of note, and in contrast with the other participants, two participants reported strongly increased pinprick sensitivity at all area at D1. This might reflect a prolonged capsaicin-induced secondary mechanical hyperalgesia that has been well described in other studies in the subacute phase after capsaicin application (Simone et al., 1989; LaMotte et al., 1991; Torebjork et al., 1992).

## Discussion

Our results confirm that the topical application of high-concentration capsaicin during 1 h induces, in healthy human volunteers, a temporary reduction of the sensitivity to short-lasting heat stimuli, which is maximal on day 1 and then progressively recovers within 1 week. In contrast, topical capsaicin had no effect on the sensitivity to short-lasting cool stimuli. Interestingly, the capsaicin-induced changes in heat sensitivity affected not only the ability to perceive high-intensity heat stimuli activating A- and/or C-fiber thermonociceptors, but also affected the ability to perceive innocuous warm sensations that are often considered to not depend on TRPV1-sensitive afferents. Most importantly, whereas the reduced sensitivity to high-intensity heat stimuli (55°C) detected with RTs compatible with the conduction velocity of Aδ-fibers was restricted to the capsaicin-treated skin, the reduced sensitivity to intermediate- and low-intensity heat stimuli detected with RTs in the C-fiber conduction range (38°C and 44°C) extended well beyond the area of capsaicin-treated skin.

### Capsaicin Desensitization Has No Effect on Cool Sensitivity

The sensitivity to short-lasting innocuous cool stimuli (10°C) was not affected by capsaicin-treatment. This is in accordance with findings of previous studies in humans and suggests a segregation within the thermal system regarding afferents responsible for sensing heating or cooling stimuli (Malmberg et al., 2004; Kennedy et al., 2010; Lo Vecchio et al., 2018). Additional evidence comes from transgenic mice models showing that the majority of neurons in the mammalian thermal system either responds to cooling or heating stimuli, and only a small fraction (~10%) is able to respond to both (Yarmolinsky et al., 2016). It is until today not clear however whether these bimodal neurons exist in humans, and if they would, whether they exist in the same proportions as in mice. Cool sensors express the TRPM8 receptor, and not TRPV1 under normal conditions (Kobayashi et al., 2005). Average RTs to cool stimuli had relatively short latencies (around 400 ms) compatible with the notion that the sensations elicited by short-lasting cool stimuli are predominantly related to the activation of cool-sensitive Aδ-fiber afferents (Campero et al., 2001; Churyukanov et al., 2012; De Keyser et al., 2018).

### Capsaicin Desensitization Affects Aδ- and C-Fiber Related Responses Differently

At day 0, the short-lasting high-intensity heat stimuli (55°C) were detected with short-latency RTs compatible with the conduction velocity of Aδ-fiber nociceptors. These high-intensity heat stimuli had a clearly pricking and unpleasant quality, typical of the so-called sensation of “first pain” elicited by phasic high-intensity heat stimuli (Dusch et al., 2016). It is generally accepted that very brief pulses of radiant heat predominantly generate responses related to the activation of type 2 heat-sensitive A-fiber nociceptors as these afferents respond quickly to rapid changes in skin temperature whereas type 1 heat-sensitive A-fiber nociceptors generate sustained activity only if they are exposed to relatively long-lasting heat stimuli (Vallbo et al., 1995; Treede et al., 1998; Magerl et al., 2001). Sensitivity to the high-intensity heat stimuli was markedly reduced at day 1. In contrast, topical capsaicin did not significantly affect sensitivity to mechanical pinprick stimuli (**Supplementary Figure S5**). This further supports the notion that the reduced sensitivity to high intensity heat stimuli was due to reduced function of type 2 heat-sensitive nociceptors. Treede et al. showed that unlike type 2 heat-sensitive nociceptors, type 1 heat-sensitive nociceptors respond strongly to mechanical pinprick stimuli. Hence, an impairment of type 1 heat-sensitive nociceptors would be expected to result in a reduced sensitivity to pinprick stimulation (Treede et al., 1995; Treede et al., 1998).

At day 0, intermediate intensity heat stimuli (44°C) and low intensity heat stimuli (38°C) were detected with long-latency RTs compatible with the conduction velocity of unmyelinated C-fibers. The qualities of the elicited sensations were markedly different from those elicited by high-intensity heat stimuli. Intermediate intensity heat stimuli were most-often qualified as burning and/or warm, reminiscent of the “second pain” sensation which is related to thermal inputs conveyed by heat-sensitive C-fiber nociceptors (Plaghki and Mouraux, 2003). Low intensity heat stimuli were simply reported as warm (Yarnitsky and Ochoa, 1991).

Most interestingly, the time course and the extent of the change in sensitivity to C-fiber-mediated heat differed from the time course and the extent of the change in sensitivity to Aδ-fiber-mediated heat. Whereas the Aδ-fiber-mediated sensitivity to high-intensity heat stimuli recovered almost completely within 1 week, C-fiber-mediated insensitivity to intermediate and low-intensity heat stimuli was longer-lasting, and still markedly present at day 7. Furthermore, the loss of sensitivity to Aδ-fiber-mediated heat was restricted to the area of treated skin whereas the loss of sensitivity to C-fiber-mediated heat spread several centimeters beyond the treated skin.

A potential explanation for the spatial spread of the capsaicin-induced reduction in sensitivity to intermediate and low-intensity heat stimuli could reside in the fact that heat-sensitive C-fibers may have larger receptive fields than heat-sensitive type 2 Aδ-fiber nociceptors. Studies in humans have suggested that Aδ-fiber nociceptors have relatively small receptive fields (2 mm^2^) (Dusch et al., 2016), and this could explain the demarcated loss of Aδ-fiber-related sensitivity to high-intensity heat stimuli restricted to the capsaicin-treated skin. Conversely, depending on the stimuli used for receptive field mapping, C-fiber afferents appear to have receptive fields varying from relatively small oval-shaped receptive fields of mechanosensitive C-units (7–15 mm^2^ depending on the strength of the tactile stimulus applied) (Wessberg et al., 2003), to larger patch-like receptive fields of mechano- and heat-sensitive polymodal C-fibers (average 106 mm^2^) (Schmelz et al., 1996; Schmidt et al., 1997) and mechano-insensitive and heat-sensitive C-fibers (60 mm^2^ as measured at the foot and leg) (Schmelz et al., 1996; Schmelz and Schmidt, 2010). C-fiber afferents having large receptive fields spanning over both the capsaicin-treated skin and the neighboring untreated skin could explain, at least in part, a reduction of heat sensitivity within the neighboring skin. Furthermore, it has been suggested that in conditions of skin inflammation or after capsaicin application, receptive fields behave dynamically and insensitive branches of mechanosensitive C-nociceptors can become responsive (Schmelz et al., 1994). Another difference between Aδ- and C-fiber afferents could be receptor density, as it has been suggested that Aδ-nociceptors are less densely distributed than C-nociceptors (Bragard et al., 1996; Rage et al., 2010). Furthermore, it is increasingly recognized that non-neuronal cells may contribute to the transduction of nociceptive stimuli applied onto the skin. Indeed, it was demonstrated that TRPV1-mediated keratinocyte stimulation can induce spinal activity, nocifensive behavior and conditioned place aversion in mice (Pang et al., 2015). Furthermore, it was recently shown that a specialized type of Schwann cells resides at the epidermal/dermal border having direct contact to epidermal free nerve ending of CGRP-, P2RX3-, and TRPV1-positive nociceptors. Interestingly, optogenetic activation of these cells led to pain-related behaviors. Besides, these cells appeared to be mechanosensitive and were responsible for the interconnection between different nociceptive fibers in the epidermis (Abdo et al., 2019). These studies suggest that non-neural cells could contribute to the extent and dynamics of the receptive fields of free nerve endings.

The spatial spread of the capsaicin-induced reduction in sensitivity to C-fiber-mediated heat could also involve changes occurring at the level of the central nervous system (Henrich et al., 2015; Kronschlager et al., 2016). For example, it is well known that intense or sustained nociceptive stimulation of the skin can induce prolonged changes in the synaptic transmission of nociceptive input extending beyond the conditioned area, explaining for example the secondary hyperalgesia that follows acute topical capsaicin or high-frequency electrical stimulation of the skin (van den Broeke and Mouraux, 2014a; van den Broeke and Mouraux, 2014b; Henrich et al., 2015; van den Broeke et al., 2019). Similarly, it is also well known that noxious stimuli delivered at one location tends to suppress the spinal transmission of nociceptive input originating from other locations, through a mechanism referred to as diffuse noxious inhibitory control.

Another central mechanism possibly explaining the remote effects of capsaicin may be activity-dependent reorganization of thalamic or S1 somatosensory maps. These changes appear to occur rapidly after peripheral denervation or local anesthesia (Pettit and Schwark, 1993; Rasmusson et al., 1993). It is thought that loss of C-fiber input caused by capsaicin application relieves tonic inhibition of synapses at central level, inducing the reorganization of excitatory receptive fields of cuneate nuclei, thalamic and S1 sensory neurons (Rasmusson et al., 1993; Pettit and Schwark, 1996; Jones, 2000) and sometimes also inducing the appearance of new inhibitory receptive fields (Pettit and Schwark, 1996). Capsaicin-induced changes could be induced both by the short but intense period of overstimulation of capsaicin-sensitive fibers during patch application, and by the later period of axonal ablation leading to a temporary deafferentation of the previously overstimulated capsaicin sensitive peripheral terminals.

It should be noted that, although participants were naïve about the long-term effects of capsaicin on thermal sensitivity, the capsaicin treatment and the fact that this sensitivity was being tested over several days could have induced some expectations on potential after-effects. Importantly, it seems unlikely that such effects of expectations could have induced changes in thermal sensitivity having different time courses according to the type of thermal stimuli and their location.

### Capsaicin Desensitization Impairs Innocuous Warmth Sensation

The sensitivity to innocuous warm stimuli (38°C) was markedly impaired after topical capsaicin treatment, and this impairment extended beyond the treated skin. This observation is in line with other studies reporting increased warm detection thresholds following capsaicin treatment (Mainka et al., 2016; Lo Vecchio et al., 2018). Yet, heat sensitivity outside the capsaicin-treated area was not investigated in these studies. Considering that, in physiological conditions, TRPV1 should not be activated by low-intensity warm stimuli, it has been suggested that the effect of capsaicin on the sensitivity to warmth could result from an interaction of TRPV1 with other TRP receptors such as TRPV3, TRPV4, and TRPM2 (Lo Vecchio et al., 2018; Jeon and Caterina, 2018). At least in mice it was shown that the complete population of warm-sensitive neurons expressed TRPV1 and that this was a population of neurons distinct from noxious heat-sensing neurons. Ablation of these neurons completely abolished sensitivity to warmth (Yarmolinsky et al., 2016). These studies and our current data suggest effects of topical capsaicin on TRPV1-positive C-fibers involved in the perception of innocuous warmth.

## Conclusion

By mapping the spatial extent and characterizing the time course of the changes in sensitivity to heat, cold and pinprick following the topical application of high-concentration capsaicin during 1 h, we show that the reported “ablation” of epidermal nerve fibers that follows this treatment is associated with (1) a selective impairment of heat sensitivity without any concomitant changes in the sensitivity to cold and (2) a differential effect on the sensitivity to thermal inputs conveyed by Aδ- and C -fibers. Most interestingly, we observed that the reduced sensitivity to Aδ-fiber-mediated heat is restricted to the capsaicin-treated skin, whereas the reduced sensitivity to C-fiber-mediated heat extends well beyond the treated skin. Moreover, the time course of the reduced sensitivity to C-fiber-mediated input was more prolonged than the time course of the reduced sensitivity to Aδ-fiber-mediated input.

## Significance Statement

We believe that our paper is of broad interest to those interested in pharmacological applications in pain research, in understanding the sensory afferents responsible for thermal and pain sensations, and in the perceptual reorganization after sensory loss and neural plasticity. Our data strengthen the view that topical capsaicin has a broader working mechanism than solely “desensitizing” capsaicin-sensitive afferents within the treated skin. Indeed, our results show that the reduced sensitivity to A-fiber-mediated heat stimuli following topical capsaicin is restricted to the capsaicin-treated skin and relatively short lasting, whereas the reduced sensitivity to C-fiber-mediated heat stimuli extends well beyond the treated skin and is longer lasting. This information, compatible with the view that the changes in thermal sensitivity following topical capsaicin may involve changes at the level of the central nervous system, is important for researchers aiming to understanding how topical capsaicin can attenuate pain in patients suffering from neuropathic pain.

## Data Availability Statement

All datasets generated for this study are included in the article/**Supplementary Material**.

## Ethics Statement

The studies involving human participants were reviewed and approved by UCLouvain commission d’éthique bio-médicale hospitalo-facultaire. The patients/participants provided their written informed consent to participate in this study.

## Author Contributions

SN: study design, data collection, data analyses, and manuscript writing. AM: supervision and manuscript writing.

## Funding

SN was supported by an internal grant for foreign post-docs “MoveIn Louvain” and by “Fondation Louvain.”

## Conflict of Interest

The authors declare that the research was conducted in the absence of any commercial or financial relationships that could be construed as a potential conflict of interest.

## Supplementary Material

Supplementary figure S1 Quality ratings in response to high intensity heat stimuli (55 °C) at remote locations of the capsaicin-treated forearm (yellow), close to the border of the capsaicin-treated skin (orange), at the capsaicin-treated skin (red), and at the contralateral forearm (purple). A. Quality ratings “Pricking”. B. Quality ratings “Pointed”. C. Quality ratings “Warm”. D. Quality ratings “Burning”. E. Quality ratings “Unpleasant”. Graphs show only quality descriptors that had been assigned ≥ 10% of total stimuli applied. Graphs represent mean ± SEM. The + indicates statistical significances compared to d0 within one location, whereas # demonstrates statistical significances between border and capsaicin location, the * between remote and capsaicin treated location, and the ° between the contralateral side and capsaicin treated area (+/*/° ≤ 0.05, ++/## ≤ 0.01 and ***/+++/### ≤ 0.001).

Supplementary figure S2 Quality ratings in response to intermediate intensity heat stimuli (44 °C) at remote locations of the capsaicin-treated forearm (yellow), close to the border of the capsaicin-treated skin (orange), at the capsaicin-treated skin (red), and at the contralateral forearm (purple).A. Quality ratings “Pricking” B. Quality ratings “Warm” C. Quality ratings “Pointed”. D. Quality ratings “Unpleasant”. Graphs show only quality descriptors that had been assigned ≥ 10% of total stimuli applied. Graphs represent mean ± SEM. The + indicates statistical significances compared to d0 within one location, whereas # demonstrates statistical significances between border and capsaicin location, the * between remote and capsaicin treated location, and the ° between the contralateral side and capsaicin treated area (+/# ≤ 0.05, and ***/+++/###/°°° ≤ 0.001).

Supplementary figure S3 Quality ratings in response to low intensity heat stimuli (38 °C) at remote locations of the capsaicin-treated forearm (yellow), close to the border of the capsaicin-treated skin (orange), at the capsaicin-treated skin (red), and at the contralateral forearm (purple). A. Quality ratings “Warm”. B. Quality ratings “Unpleasant”. Graphs show only quality descriptors that had been assigned ≥ 10% of total stimuli applied. Graphs represent mean ± SEM. The + indicates statistical significances compared to d0 within one location, whereas # demonstrates statistical significances between border and capsaicin location, the * between remote and capsaicin treated location, and the ° between the contralateral side and capsaicin treated area (+ ≤ 0.05, ++/°° ≤ 0.01 and ***/ +++/###/°°° ≤ 0.001).

Supplementary figure S4 Quality ratings in response to cooling stimuli (10 °C) at remote locations of the capsaicin-treated forearm (yellow), close to the border of the capsaicin-treated skin (orange), at the capsaicin-treated skin (red), and at the contralateral forearm (purple). A. Quality ratings “Cool”. B. Quality ratings “Wet”. C. Quality ratings “Burning”. D. Quality ratings “Unpleasant”. Graphs show only quality descriptors that had been assigned ≥ 10% of total stimuli applied. Graphs represent mean ± SEM.

Supplementary figure S5 Sensitivity to pinprick stimuli (128 mN) at remote locations of the capsaicin700 treated forearm (yellow), close to the border of the capsaicin-treated skin (orange), at the capsaicintreated skin (red), and at the contralateral forearm (purple). A. Intensity ratings. B. Non-perceived stimuli. C. Quality ratings “Touch”. D. Quality ratings “Pricking”. E. Quality ratings “Pointed”. F. Quality ratings “Unpleasant”. Graphs show only quality descriptors that had been assigned ≥ 10% of total stimuli applied. Graphs represent mean ± SEM.
