# Supplementary figures and images for: Capsaicin-Induced Skin Desensitization Differentially Affects A-Delta and C-Fiber-Mediated Heat Sensitivity
Source: Front Pharmacol. 2020 May 19;11:615. doi: 10.3389/fphar.2020.00615 (PMC7248294; doi:10.3389/fphar.2020.00615)

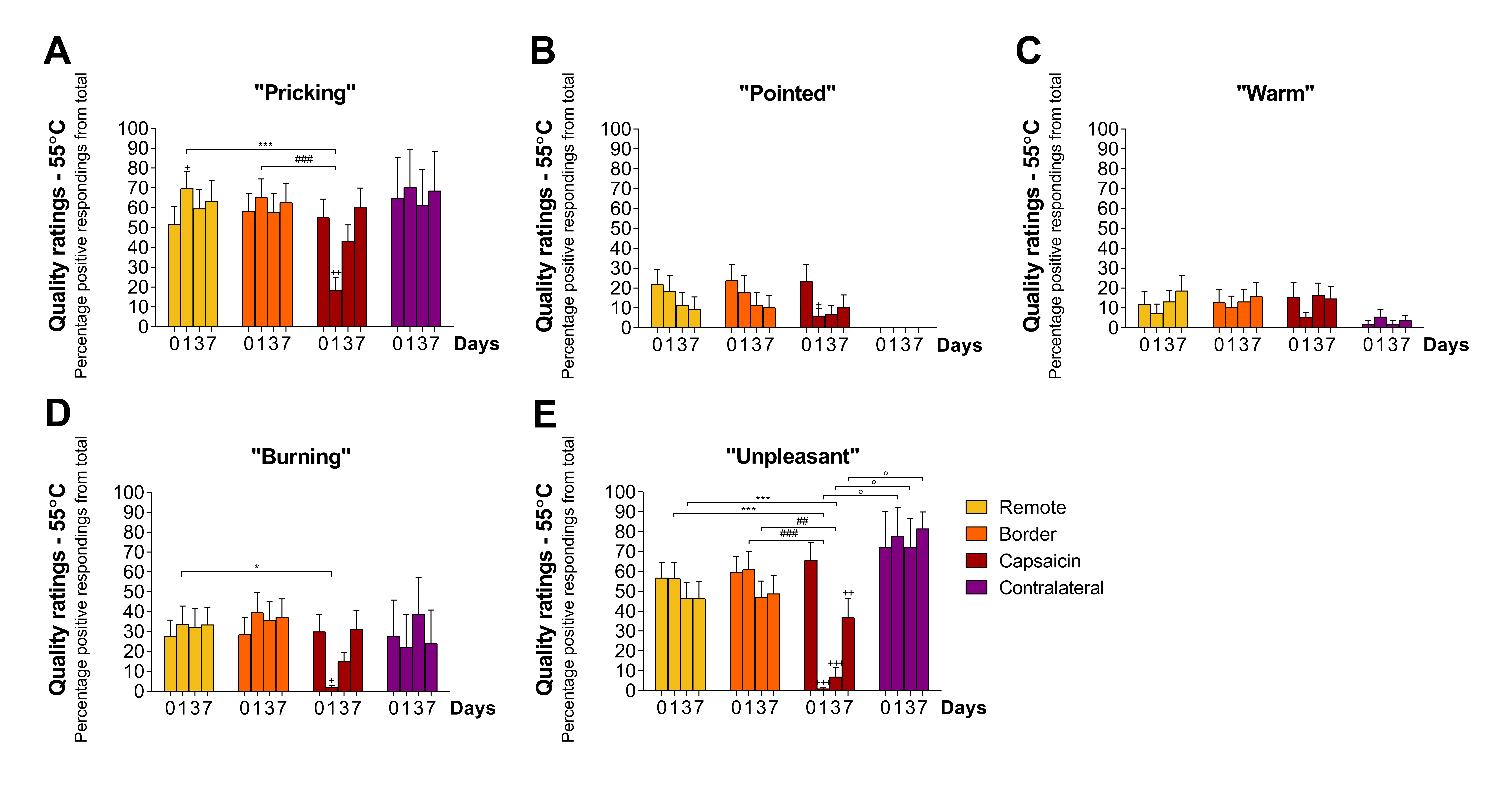

Supplement: Supplementary figure S1 — Quality ratings in response to high intensity heat stimuli (55 °C) at remote locations of the capsaicin-treated forearm (yellow), close to the border of the capsaicin-treated skin (orange), at the capsaicin-treated skin (red), and at the contralateral forearm (purple). A. Quality ratings “Pricking”. B. Quality ratings “Pointed”. C. Quality ratings “Warm”. D. Quality ratings “Burning”. E. Quality ratings “Unpleasant”. Graphs show only quality descriptors that had been assigned ≥ 10% of total stimuli applied. Graphs represent mean ± SEM. The + indicates statistical significances compared to d0 within one location, whereas # demonstrates statistical significances between border and capsaicin location, the * between remote and capsaicin treated location, and the ° between the contralateral side and capsaicin treated area (+/*/° ≤ 0.05, ++/## ≤ 0.01 and ***/+++/### ≤ 0.001). [file Image_1.tif]

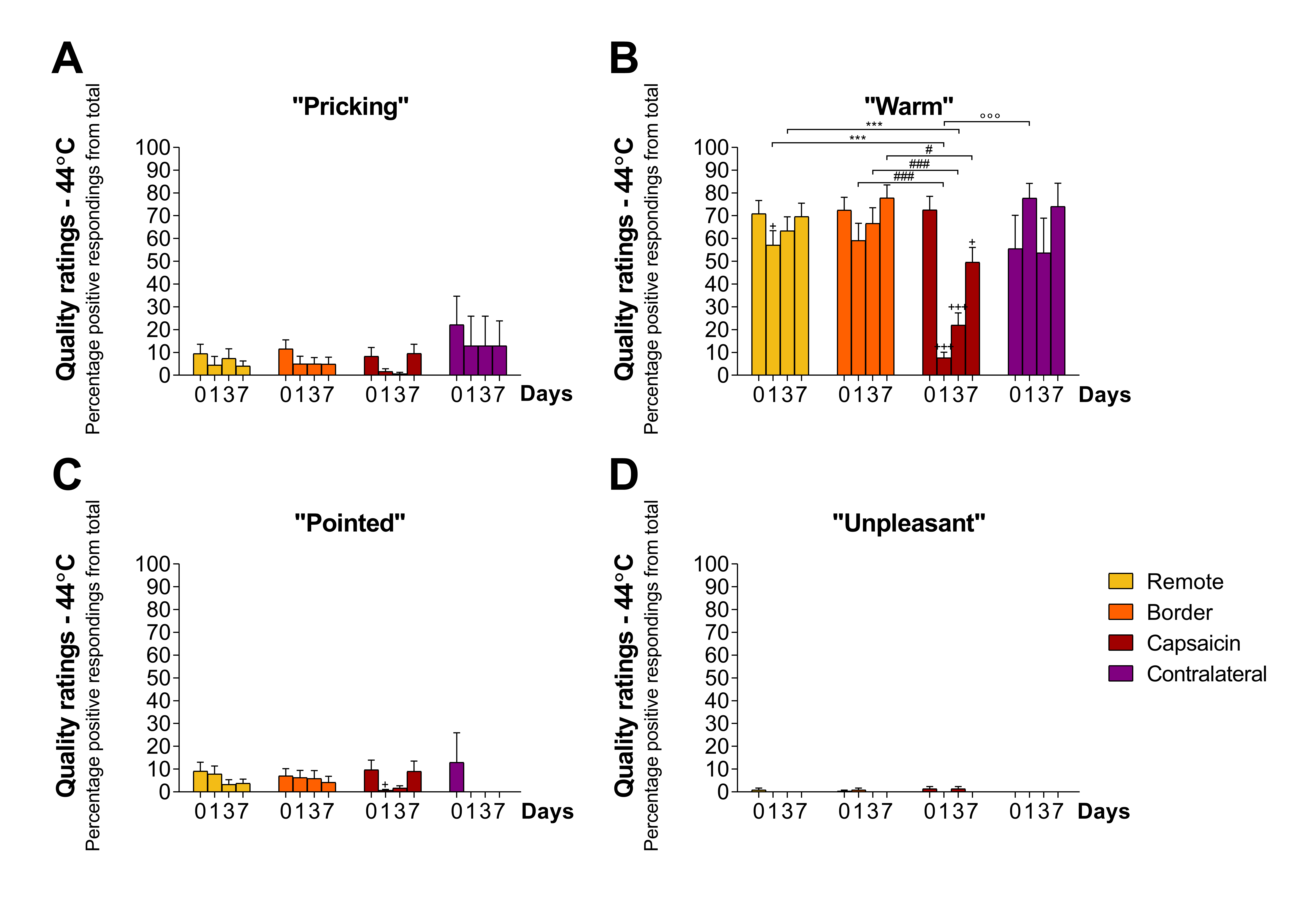

Supplement: Supplementary figure S2 — Quality ratings in response to intermediate intensity heat stimuli (44 °C) at remote locations of the capsaicin-treated forearm (yellow), close to the border of the capsaicin-treated skin (orange), at the capsaicin-treated skin (red), and at the contralateral forearm (purple).A. Quality ratings “Pricking” B. Quality ratings “Warm” C. Quality ratings “Pointed”. D. Quality ratings “Unpleasant”. Graphs show only quality descriptors that had been assigned ≥ 10% of total stimuli applied. Graphs represent mean ± SEM. The + indicates statistical significances compared to d0 within one location, whereas # demonstrates statistical significances between border and capsaicin location, the * between remote and capsaicin treated location, and the ° between the contralateral side and capsaicin treated area (+/# ≤ 0.05, and ***/+++/###/°°° ≤ 0.001). [file Image_2.tif]

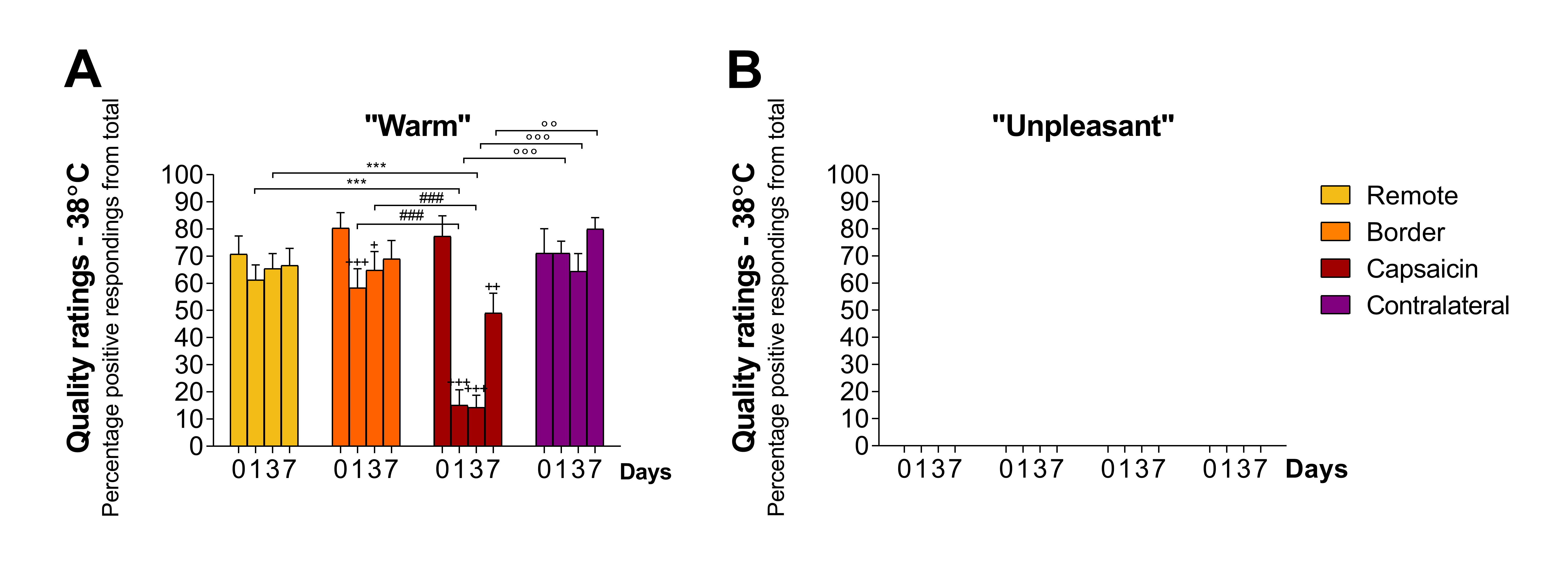

Supplement: Supplementary figure S3 — Quality ratings in response to low intensity heat stimuli (38 °C) at remote locations of the capsaicin-treated forearm (yellow), close to the border of the capsaicin-treated skin (orange), at the capsaicin-treated skin (red), and at the contralateral forearm (purple). A. Quality ratings “Warm”. B. Quality ratings “Unpleasant”. Graphs show only quality descriptors that had been assigned ≥ 10% of total stimuli applied. Graphs represent mean ± SEM. The + indicates statistical significances compared to d0 within one location, whereas # demonstrates statistical significances between border and capsaicin location, the * between remote and capsaicin treated location, and the ° between the contralateral side and capsaicin treated area (+ ≤ 0.05, ++/°° ≤ 0.01 and ***/ +++/###/°°° ≤ 0.001). [file Image_3.tif]

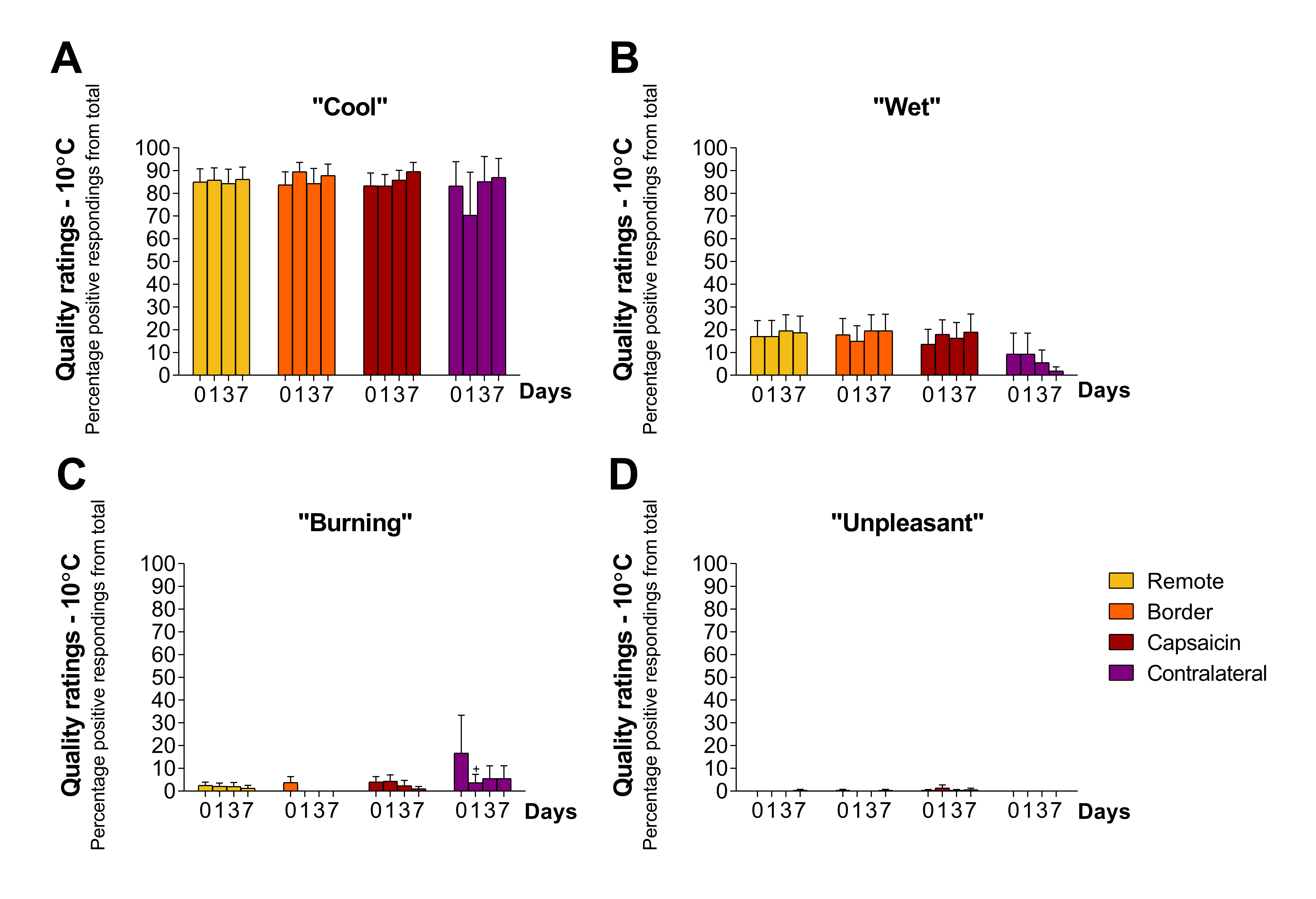

Supplement: Supplementary figure S4 — Quality ratings in response to cooling stimuli (10 °C) at remote locations of the capsaicin-treated forearm (yellow), close to the border of the capsaicin-treated skin (orange), at the capsaicin-treated skin (red), and at the contralateral forearm (purple). A. Quality ratings “Cool”. B. Quality ratings “Wet”. C. Quality ratings “Burning”. D. Quality ratings “Unpleasant”. Graphs show only quality descriptors that had been assigned ≥ 10% of total stimuli applied. Graphs represent mean ± SEM. [file Image_4.tif]
